# Gender and racial/ethnic disparities in the life-course timing of first unpaid caregiving: a survival analysis approach

**DOI:** 10.1093/geronb/gbag165

**Published:** 2026-08-18

**Authors:** Chioun Lee, Dana A Glei, Soojin Park, Maxine Weinstein

**Affiliations:** Department of Sociology, University of California, Riverside, Riverside, California, United States; Center for Population and Health, Georgetown University, Washington, District of Columbia, United States; School of Education, University of California, Riverside, Riverside, California, United States; Center for Population and Health, Georgetown University, Washington, District of Columbia, United States; (Social Sciences Section)

**Keywords:** Unpaid caregiving, Life-course timing, Survival analysis, Gender differences, Racial/ethnic disparities

## Abstract

**Objectives:**

Extensive research documents gender and racial/ethnic disparities in unpaid caregiving, defined as providing informal care to adults or children with illness, disability, or special care needs, excluding routine childcare. Yet, little is known about how the timing of caregiving entry varies across diverse groups. Despite the growing number of foreign-born Hispanics in the United States, evidence on caregiving entry among Hispanic subgroups remains scarce.

**Methods:**

We used data (*N *= 11,624) from the Health and Retirement Study (1992–2020) and the Life History Mail Survey (2017 and 2019). Using survival analysis, we estimated how the risk of entering first unpaid caregiving (lasting 6 months or longer) unfolds over the life course and varies by gender and racial/ethnic groups, as well as nativity among Hispanics.

**Results:**

The likelihood of entering caregiving increased gradually with age, peaking around age 56 and declining thereafter. Women were roughly twice as likely as men to become caregivers, with the gender gap widening through midlife and persisting into old age. Differences between non-Hispanic Blacks and non-Hispanic Whites were minimal. In contrast, U.S.-born Hispanics entered caregiving earlier in the life course than non-Hispanic Whites, whereas foreign-born Hispanics showed reduced entry particularly across midlife and old age. U.S.-born Hispanics show higher entry into caregiving than foreign-born Hispanics, with differences emerging in young adulthood.

**Discussion:**

Our findings underscore persistent gender disparities in unpaid caregiving beginning early in adulthood and continuing into later life. The distinct timing patterns among Hispanic subgroups highlight the importance of considering nativity status in caregiving research.

Unpaid caregiving, defined in this study as providing informal care to adults or children with illness, disability, or special care needs, excluding routine childcare, is an important form of family caregiving in the United States (National Alliance for Caregiving [NAC] & AARP, 2025). Within a life-course framework, the consequences of unpaid caregiving may differ depending on the timing of entry into this role. Life transitions are considered normative or non-normative depending on the age at which they occur (Settersten & Hägestad, 1996). A life transition can yield contrasting consequences depending on its timing within an individual’s developmental stage (Elder, 1994) and may trigger disadvantages that accumulate over time (Dannefer, 2003). Caregiving responsibilities during midlife often align with the culturally expected timeline of major family obligations. In contrast, early-entry caregiving for family members with special health needs may disrupt normative life-course sequences across domains such as educational attainment (Miller et al., 2024), work and financial independence (NAC & AARP, 2015), and intimate relationships (King McLaughlin et al., 2019), with lasting implications for later life. Although gendered and racial/ethnic disparities in caregiving exposure are well documented (Pinquart & Sörensen, 2006), we know little about how the onset of unpaid caregiving unfolds differently across these groups over the life course.

Prior work documents a clear gender gap in unpaid caregiving to ill or aging family members in midlife and old age (Pinquart & Sörensen, 2006), with middle-aged women providing care at substantially higher rates than their male counterparts. However, the gender gap in certain forms of caregiving may narrow in old age, as men become more likely to become spousal caregivers (Glauber, 2017). By contrast, evidence on young adulthood or earlier is mixed. Some U.S.-based studies report more female caregivers among adolescents aged 15–18, but no gender differences in young adults aged 19–22 (Miller et al., 2024), and even slightly higher prevalence among men than women aged 18–25 (Levine et al., 2005). In contrast, a European study finds higher caregiving rates among women than men aged 25–29 (Di Gessa et al., 2022). Despite this growing literature, prior work has focused exclusively on prevalence differences at specific life stages. What remains unanswered is when gender differences in caregiving first emerge and how the gender gap unfolds across the life-course.

Race and ethnicity are additional elements in the variations of caregiving involvement in the United States. Racial/ethnic differences in some aspects of caregiving are well-known. Compared with non-Hispanic Whites, non-Hispanic Blacks and Hispanics are more likely to provide informal care, spend more hours, and deliver more intensive care to family members or others with illness or disability (NAC & AARP, 2025). These racialized patterns reflect a combination of sociodemographic and structural factors, including higher prevalence of chronic conditions and disability in minority communities (Quiñones et al., 2019), limited access to paid leave or affordable long-term care options, and a greater likelihood of co-residing with care recipients (NAC & AARP, 2025). Cultural norms emphasizing strong family ties, mutual obligation, and interdependence, particularly salient in Hispanic communities, further reinforce expectations surrounding caregiving roles (Dilworth-Anderson et al., 2002).

With the growing diversity among older adults, the immigration status of caregivers, particularly within Hispanic populations, plays a critical role in caregiving patterns, as nativity status (i.e., U.S-born vs. foreign-born) can affect geographic proximity to care recipients (Miyawaki & Hooyman, 2023), the size and composition of social and caregiving networks, and access to formal care options (Rote & Moon, 2018). It may also contribute to differences in caregiving demands: U.S.-born Hispanics experience higher rates of morbidity and mortality than their foreign-born counterparts due to diminished immigrant health advantages and lifetime exposure to structural and behavioral risks (Fenelon et al., 2017; Garcia et al., 2018).

It has long been suggested that Hispanic and Black caregivers enter caregiving roles for family members with health-related needs earlier in life than non-Hispanic Whites (Aranda & Knight, 1997; Fingerman et al., 2025). However, few empirical studies, particularly those using population-based data, have systematically investigated this hypothesized pattern. A recent study using the American Time Use Survey (2013–2019) found that non-Hispanic White youth are less likely to provide care than Black and Hispanic youth, suggesting earlier entry into caregiving among minorities, but this evidence is based primarily on caregiving prevalence among adolescents aged 15 to 18 (Miller et al., 2024). More importantly, no study has examined how the timing of caregiving varies depending on nativity within Hispanic populations.

Importantly, recent work highlights the significant role of caregiving in shaping gender differences and racial disparities (particularly among women) in later-life health (Lee et al., 2026b). Consistent with the principle of the *timing of lives* (Elder, 1994), emerging evidence suggests the long-term consequences of caregiving depend on when it occurs in the life course. Age at entry into caregiving remains significantly associated with later-life health outcomes, even after accounting for other caregiving characteristics, such as duration (Lee et al., 2026a). These findings underscore the importance of examining how the timing of caregiving onset varies across gender and race/ethnicity, as such differences may contribute to the emergence and persistence of health disparities over the life course.

## Current study

Using survival analysis with rich life-course caregiving histories from the HRS, we investigate a long-standing unanswered question: whether the timing of first unpaid caregiving varies by gender and racial/ethnic group, and how such differences unfold across the life course. We hypothesize that women experience earlier entry and higher rates of caregiving across all life stages than men (H1). Building on prior work on young caregivers, we hypothesize that, relative to non-Hispanic Whites, non-Hispanic Blacks and Hispanics are more likely to enter unpaid caregiving at earlier ages (H2). Finally, considering the role of immigration-related factors that may affect caregiving demands, we hypothesize that among Hispanics, U.S.-born individuals begin caregiving earlier than their foreign-born counterparts (H3).

## Method

### Sample

We used data from the Health and Retirement Study (HRS; 1992–2020) and the Life History Mail Survey (LHMS; 2017 and 2019, Larkina et al., 2025). The LHMS is an off-wave supplement to HRS that collects retrospective information on life-course experiences, including caregiving histories (for details, see Supplementary Text 1). The analytic sample was restricted to respondents born in 1965 or earlier (late Boomers or older) who completed the LHMS and reported caregiving information. We further limited the sample to non-Hispanic White, non-Hispanic Black, or Hispanic. The final sample included 11,624 individuals, 26% of whom reported providing unpaid caregiving (*N* = 3,073). For additional details, see Supplementary Figure 1 and Text 2.

### Measures

*Age at first unpaid caregiving* was assessed using the LHMS life history calendar, in which respondents reported whether they had ever provided unpaid care for 6 months or longer to a relative or friend with special needs, such as illness, disability, or mental health problems, excluding routine childcare for children without special needs. Caregiving tasks include a range of activities, such as personal care, assistance with household tasks, financial management, arranging medical services, and regular check-ins. Respondents indicated the start and end years of caregiving for up to five caregiving episodes. The age at first unpaid caregiving was calculated by subtracting the respondent’s birth year from the start year of their earliest caregiving episode.

Covariates include demographic variables (age, gender, race/ethnicity, nativity, birth cohort, number of siblings) that may influence selection into caregiving. *Age* was measured when respondents participated in the LHMS interview. *Gender* is based on self-reported sex (female vs. male). Race/ethnicity and nativity (U.S.-born vs. foreign-born) were self-reported. Using these variables, *race/ethnicity* was categorized as non-Hispanic White, non-Hispanic Black, U.S.-born Hispanic, and foreign-born Hispanic. Because foreign-born non-Hispanic Whites and Blacks comprised less than 5% of their respective groups, we did not differentiate Whites and Blacks by nativity. *Birth cohorts* were grouped into three categories: (1) HRS or earlier cohorts (born ≤1941), (2) War Babies/Early Boomers (born 1942–1953), and (3) Mid/Late Boomers (born 1954–1965). The *number of siblings* was measured as the maximum reported across all HRS waves and was top-coded at seven or more.

### Analytic strategy

We used survival analysis to estimate the risk of first caregiving exposure. Age served as the time metric, with a minimum caregiving entry age set at 10, resulting in an analytic age range from 10 to 89. The lower bound of age 10 was based on prior research on young caregivers (Lacey et al., 2022) and the caregiving tasks captured in the LHMS (e.g., assistance with household tasks; see Supplementary Text 3 for details). Respondents who never provided caregiving were censored at their age at the LHMS interview (right-censoring, *n* = 8,551).

Survival analyses proceeded in two stages. First, we used a nonparametric life table (Nelson–Aalen estimator) to estimate gender and racial/ethnic disparities in proportions of individuals who have experienced first caregiving by age *t*, beginning with age 10 and presented at 10-year intervals through age 80. We compare whether the cumulative risk differs across groups. Statistical significance was assessed using non-overlapping confidence intervals based on the Goldstein–Healy rule (Goldstein & Healy, 1995). We used *α* = 0.05 for gender comparisons, but *α* = 0.10 for racial/ethnic comparisons due to smaller sample sizes among racial/ethnic groups. To aid interpretation, we calculated the relative risk ratio at age *t* by dividing the risk for each comparison group by that for the reference group.

Second, we estimated semiparametric Cox hazards models to assess gender and racial/ethnic disparities in the risk of exposure to first caregiving, adjusted for covariates. The age-specific hazard functions significantly differed across racial/ethnic groups; thus, we added interaction terms between race/ethnicity and age to allow for non-proportional hazards. We then plotted smoothed hazard functions derived from these models to illustrate how the risk of first caregiving exposure evolves across the life course and varies by group. Because the LHMS was administered to selected HRS subsamples and individual-level sampling weights specific to the LHMS are not currently available, analyses were conducted without survey weights.

## Results

Descriptive statistics and characteristics of caregiving across groups are presented in Supplementary Tables 1 and 2. Age-specific hazard of the first caregiving is displayed in Supplementary Figure 2. Figure 1A and Table 1 show women were about twice as likely as men to become caregivers over the life course, with the gender gap widening from young adulthood (by age 20: 0.5% men vs. 0.9% women) through midlife (by age 50: 9.1% men vs. 18.1% women) and continuing into older ages (by age 70: 20.3% men vs. 40.7% women). Figure 1B shows that age-specific hazard functions of caregiving increased steadily with age for both men and women, peaked at approximately age 56, and declined thereafter.

**Figure 1:**
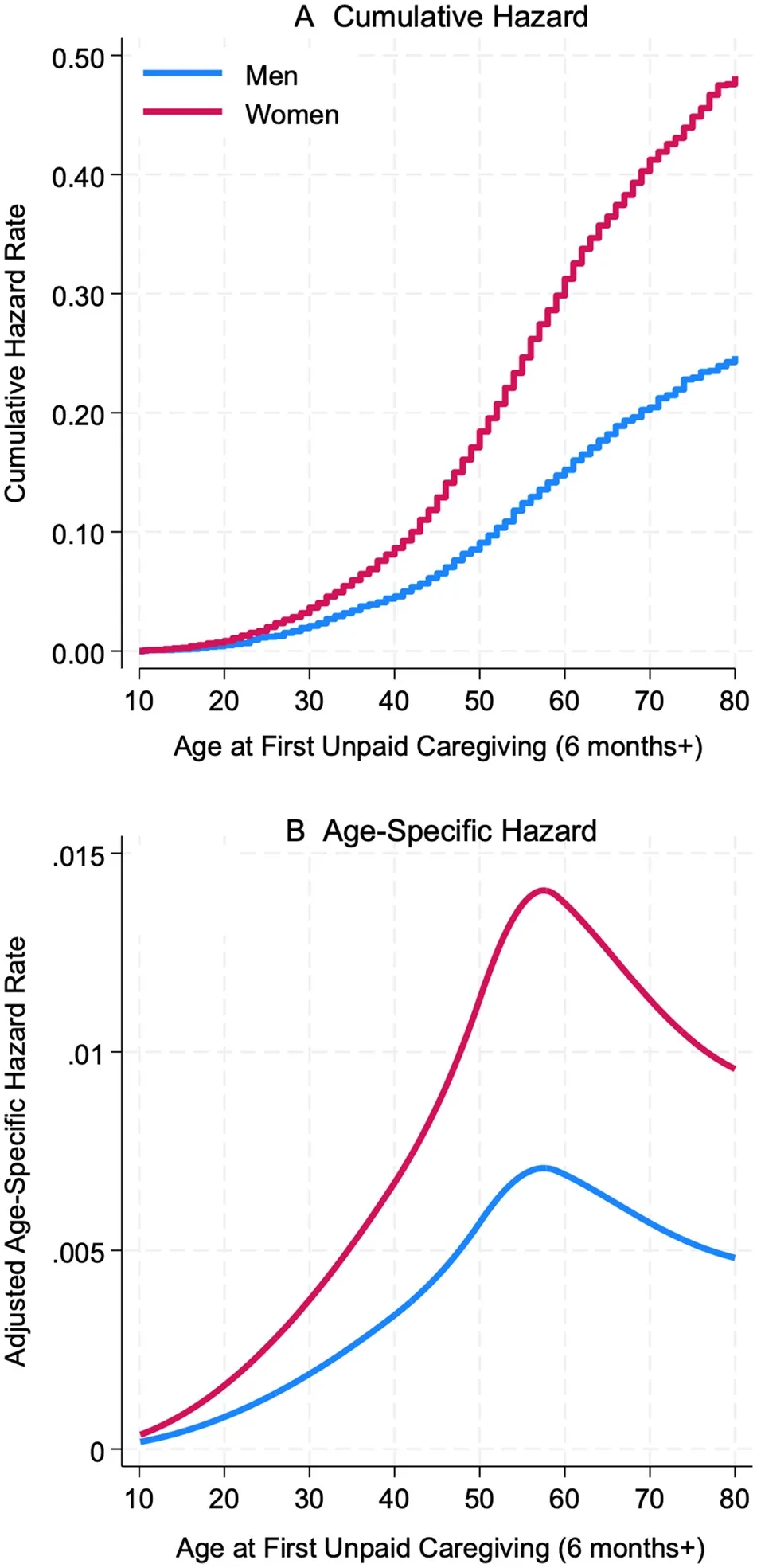
Risk of 1st unpaid caregiving over the life course by gender. (A) Cumulative hazard is estimated using the Nelson–Aalen estimator. (B) Age-specific hazard estimates are derived from Cox models controlling for gender, race/ethnicity, birth cohort, age at the Life History Mail Survey (LHMS), and the maximum number of siblings observed across the Health and Retirement Study waves (1992–2020). LHMS (2017 and 2019) = 11,624.

**Table 1:** Cumulative risk by age *t* of 1st unpaid caregiving by group (*N *= 11,624).

Age at *t*	Cumulative risk (CI)						Ratio of cumulative risk relative to reference group				
	Men	Women	Non-Hispanic White	Non-Hispanic Black	U.S.-born Hispanic	Foreign-born Hispanic	Women^a^	Non-Hispanic White^b^	U.S.-born Hispanic^b^	Foreign-born Hispanic^b^	U.S.-born Hispanic^c^
10	0.0002	0.0006	0.0005	NA	NA	0.0011	3.00	NA	NA	NA	2.20
	(0.0001–0.0008)	(0.0003–0.0012)	(0.0003–0.0009)			(0.0003–0.0041)					
20	0.005	0.009	0.007	0.008	0.010	0.008	1.67**	1.09	1.48	1.16	1.25
	(0.004–0.006)	(0.007–0.010)	(0.006–0.008)	(0.005–0.010)	(0.006–0.017)	(0.005–0.013)					
30	0.021	0.037	0.028	0.035	0.040	0.029	1.71**	1.23	1.41	1.02	1.38
	(0.019–0.025)	(0.033–0.040)	(0.026–0.031)	(0.030–0.041)	(0.031–0.052)	(0.022–0.038)					
40	0.047	0.085	0.067	0.074	0.092	0.061	1.83**	1.10	1.38*	0.91	1.51*
	(0.042–0.051)	(0.080–0.090)	(0.063–0.071)	(0.066–0.082)	(0.077–0.110)	(0.051–0.073)					
50	0.091	0.181	0.143	0.156	0.144	0.112	1.99**	1.09	1.01	0.79*	1.29
	(0.085–0.097)	(0.174–0.189)	(0.137–0.149)	(0.145–0.168)	(0.125–0.166)	(0.098–0.128)					
60	0.152	0.308	0.247	0.246	0.236	0.162	2.03**	0.99	0.95	0.65*	1.46*
	(0.144–0.160)	(0.298–0.318)	(0.240–0.255)	(0.231–0.261)	(0.210–0.265)	(0.144–0.182)					
70	0.203	0.407	0.327	0.328	0.293	0.208	2.00**	1.00	0.89	0.64*	1.41*
	(0.193–0.214)	(0.394–0.420)	(0.318–0.337)	(0.309–0.349)	(0.261–0.329)	(0.185–0.234)					
80	0.245	0.475	0.386	0.384	0.326	0.241	1.94**	1.00	0.85	0.62*	1.35*
	(0.232–0.258)	(0.460–0.491)	(0.374–0.397)	(0.359–0.411)	(0.288–0.369)	(0.208–0.278)					
*N* at risk at age 10	4,816	6,808	8,009	2,153	588	874					

*Notes.* CI = confidence interval; NA = not applicable due to zero events within specific groups. Cumulative hazard at age *t* is estimated using the Nelson–Aalen estimator. * indicates significant differences from the reference group. Reference groups:

a Men for gender,

b Non-Hispanic Whites for race/ethnicity groups,

c Foreign -born Hispanics for U.S. born Hispanics. Significance is determined by non-overlapping confidence intervals (in parentheses) under the Goldstein–Healy rule.

* *p <* .05.

** *p < .*10.

Non-Hispanic Black adults showed a higher rate than non-Hispanic Whites of caregiving entry during young adulthood (by age 30, 1.23 times), but the Black–White difference was not statistically significant. From midlife through older ages, the Black–White ratio of cumulative risk is close to 1 (Table 1), and the cumulative hazard curve and age-specific hazard function between the two groups overlapped (Figure 2).

**Figure 2:**
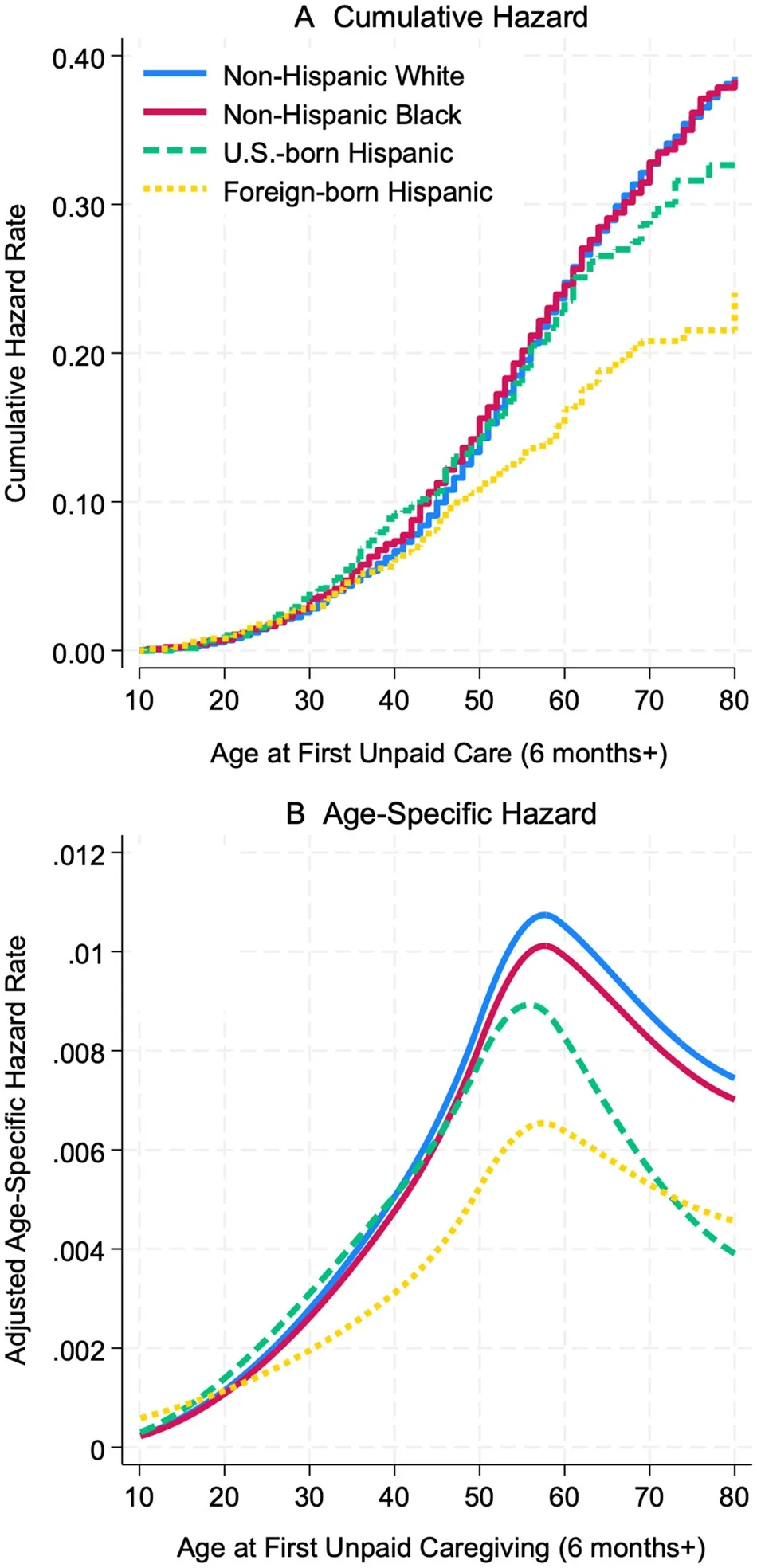
Risk of 1st unpaid caregiving over the life course by race/ethnicity. (A) Cumulative hazard is estimated using the Nelson–Aalen estimator. (B) Age-specific Hazard estimates are derived from Cox models controlling for gender, race/ethnicity, birth cohort, age at the Life History Mail Survey (LHMS), and the maximum number of siblings observed across HRS waves (1992–2020). To allow for non-proportional hazards, the model includes interactions between age and race/ethnicity.LHMS (2017 and 2019) =11,624.

In contrast, U.S.-born Hispanics were more likely than non-Hispanic Whites to begin caregiving by young adulthood. By age 40, the cumulative risk of first caregiving was 1.38 times higher for U.S.-born Hispanics (9.2%) than for non-Hispanic Whites (6.7%). However, this gap narrowed at older ages because non-Hispanic Whites were more likely than U.S.-born Hispanics to enter caregiving during midlife and beyond (Figure 2B). Foreign-born Hispanics, by comparison, showed consistently lower risks of entering caregiving, particularly midlife through old age, relative to non-Hispanic Whites.

Within the Hispanic population, U.S.-born Hispanics had higher risks of caregiving exposure than foreign-born Hispanics, with differences emerging from young adulthood (by age 40: 9.2% vs. 6.1%) and persisting into later life (by age 80: 33% vs. 24%). This pattern likely reflects earlier and more consistent caregiving entry among U.S.-born Hispanics, although age-specific hazards converged somewhat after age 70 (Figure 2B).

### Sensitivity analysis

Parents or parents-in-law were the most common first care recipients (58%; Supplementary Table 2); thus, sensitivity analyses focused on parental caregiving onset (see Supplementary Text 4). Gender differences remained consistent with the main findings (Supplementary Figures 3 and 5). In Black–White comparisons, cumulative risks did not differ significantly by age 50, but by age 60 the risk was lower for non-Hispanic Blacks, reflecting later-life acceleration in caregiving entry among non-Hispanic Whites. Differences between non-Hispanic Whites and Hispanics emerged earlier, with significant gaps by age 30 (1.75 times; Supplementary Table 3). Within Hispanics, U.S.-born Hispanics were about twice as likely as foreign-born Hispanics to initiate parental caregiving, with differences emerging around age 30 and persisting through age 60 (Supplementary Tables 3 and 4; Supplementary Figures 4 and 6).

## Discussion

Our findings reveal distinct patterns. First, we found robust and persistent gender disparities in caregiving entry, supporting our first hypothesis. Women were about twice as likely as men to begin caregiving, with a gap emerging in young adulthood, widening through midlife, and persisting into old age. The observed patterns are consistent with prior research on gendered life course caregiving (Moen et al., 1994). Our findings, however, should be interpreted in the context of intergenerational support and caregiving attitudes among baby boomers or older cohorts. Recent studies indicate that gender gaps in caregiving may have narrowed in younger cohorts (Miller et al., 2024), although gender disparities remain, particularly for intensive and primary care roles (Cha & Ailshire, 2026).

Our findings partially support the second hypothesis concerning earlier caregiving entry among racial/ethnic groups and fully support the third hypothesis regarding nativity differences among Hispanics. U.S.-born Hispanics emerged as the most vulnerable group in terms of early entry into caregiving, exhibiting a higher risk than both non-Hispanic Whites and foreign-born Hispanics. In contrast, foreign-born Hispanics appeared to be the most delayed group, with consistently lower risks of caregiving entry across the life course; their likelihood of entering caregiving was lower than U.S.-born Hispanics at most ages and remained lower than that of non-Hispanic Whites from midlife through old age. By comparison, we found little evidence of a Black–White difference in early caregiving.

The observed patterns among Hispanics align with prior bereavement research showing that, compared with non-Hispanic Whites, foreign-born Hispanics experience lower risks of family members’ deaths (e.g., mothers’ deaths) across the life course, whereas U.S.-born Hispanics exhibit higher risks, particularly in young adulthood (Donnelly et al., 2023). Lower caregiving entry among foreign-born Hispanics may reflect a combination of the healthy immigrant effect and geographic distance from parents or other family members who remain in the country of origin. In contrast, earlier onset and higher prevalence of disability among U.S.-born Hispanics (Pebley et al., 2021) may increase caregiving entry in young adulthood while reducing the likelihood of entering caregiving in old age.

The absence of Black–White differences in the timing of caregiving entry should be interpreted with caution. Several factors may contribute to this pattern. Black Americans tend to experience the loss of family members more often and at younger ages compared to non-Hispanic White Americans (Umberson et al., 2017). Among Black Americans, providing assistance to aging kin is often viewed as a normative family role rather than a distinct caregiving activity (Taylor et al., 2013). They are also more likely to transition out of caregiving roles over time (McCann et al., 2004), possibly because caregiving responsibilities are more frequently shared across extended and fictive kin networks (Taylor et al., 2013). These patterns may result in shorter or more intermittent episodes which cannot be captured in LHMS. Financial constraints may further limit the ability to sustain longer caregiving spells; families with fewer resources may be more likely to increase paid work hours rather than continue unpaid caregiving (Macchioni Giaquinto et al., 2022). In addition, selective attrition cannot be ruled out, as Black respondents and those in poorer health are less likely to participate in follow-up surveys. Taken together, these factors may attenuate observable Black–White differences in the timing of caregiving entry, particularly for caregiving episodes lasting 6 months or longer.

Our findings should be interpreted in light of some limitations. First, caregiving prevalence depends on its definition; the LHMS captures only unpaid caregiving lasting 6 months or longer, thus excluding those who provided short-term caregiving. Moreover, it does not include detailed information on caregiving (e.g., intensity, primary versus secondary roles, or caregiving network size). However, these features typically develop after caregiving begins, and age at caregiving onset is inversely related to caregiving duration (see Supplementary Text 5). Therefore, they are unlikely to introduce substantial bias in models of caregiving entry. Second, although life history calendars reduce recall bias (Axinn et al., 1999), particularly for salient events (Kreyenfeld & Bastin, 2016), retrospective reporting may still be subject to some recall error (see Supplementary Text 6 for details). In addition, the data lack detailed information on care recipients (e.g., country of origin, place of residence, and geographic proximity), which may influence the timing and likelihood of caregiving entry. Finally, although morbidity patterns (possible caregiving demands) vary among Hispanic subpopulations by country of origin, small sample sizes precluded subgroup analyses.

Despite the limitations, our study advances life-course research by documenting persistent gender disparities in caregiving entry that emerge early in adulthood and endure into later life, as well as racial/ethnic differences in timing, including distinct patterns by nativity among Hispanics. Given the central role of the timing of lives (Elder, 1994), future research should examine how unequal timing of caregiving onset contributes to persistent gender and racial/ethnic disparities in later-life health through subsequent life-course pathways, including employment, financial security, family formation, and social networks.

## Supplementary material

Supplementary material is available at *The Journals of Gerontology, Series B: Psychological Sciences and Social Sciences* online.

## Supplementary Material

gbag165_Supplementary_Data

## Data Availability

The analyses reported in this manuscript were not preregistered. The data from the Health and Retirement Study (HRS) can be accessed at https://hrs.isr.umich.edu/data-products/. We used data from the HRS core surveys (1992–2020) and the 2017 and 2019 Life History Mail Surveys.

## References

[gbag165-B1] Aranda M. P. , Knight B. G. (1997). The influence of ethnicity and culture on the caregiver stress and coping process: A sociocultural review and analysis. The Gerontologist, 37, 342–354. 10.1093/geront/37.3.342 9203758

[gbag165-B2] Axinn W. G. , Pearce L. D., Ghimire D. (1999). Innovations in life history calendar applications. Social Science Research, 28, 243–264. 10.1006/ssre.1998.0641

[gbag165-B3] Cha H. , Ailshire J. (2026). Male spousal caregivers are increasing, but only in less demanding care roles. Journal of Marriage and Family, 88, 584–595. 10.1111/jomf.70030 PMC13568684 42730112

[gbag165-B4] Dannefer D. (2003). Cumulative advantage/disadvantage and the life course: Cross-fertilizing age and social science theory. The Journals of Gerontology, Series B: Psychological Sciences and Social Sciences, 58, S327–S337. 10.1093/geronb/58.6.S327 14614120

[gbag165-B5] Di Gessa G. , Xue B., Lacey R., McMunn A. (2022). Young adult carers in the UK: New evidence from the UK Household Longitudinal Study. International Journal of Environmental Research and Public Health, 19, 14076. 10.3390/ijerph192114076 PMC9656039 36360950

[gbag165-B6] Dilworth-Anderson P. , Williams I. C., Gibson B. E. (2002). Issues of race, ethnicity, and culture in caregiving research: A 20-year review (1980–2000). The Gerontologist, 42, 237–272. 10.1093/geront/42.2.237 11914467

[gbag165-B7] Donnelly R. , Garcia M. A., Cha H., Hummer R. A., Umberson D. (2023). Exposure to family member deaths across the life course for Hispanic individuals. Demography, 60, 539–562. 10.1215/00703370-10604036 PMC10233821 36920950

[gbag165-B8] Elder G. H. Jr. (1994). Time, human agency, and social change: Perspectives on the life course. Social Psychology Quarterly, 57, 4–15. 10.2307/2786971

[gbag165-B9] Fenelon A. , Chinn J. J., Anderson R. N. (2017). A comprehensive analysis of the mortality experience of Hispanic subgroups in the United States: Variation by age, country of origin, and nativity. SSM—Population Health, 3, 245–254. 10.1016/j.ssmph.2017.01.011 PMC5769052 29349222

[gbag165-B10] Fingerman K. L. , Zhou Z., Haley W. E., Zarit S. H. (2025). Young adult caregivers for older family members: Setting a new research agenda. Innovation in Aging, 9, igae112. 10.1093/geroni/igae112 PMC11995456 40231160

[gbag165-B11] Garcia M. A. , Garcia C., Chiu C.-T., Raji M., Markides K. S. (2018). A comprehensive analysis of morbidity life expectancies among older Hispanic subgroups in the United States: Variation by nativity and country of origin. Innovation in Aging, 2, igy014. 10.1093/geroni/igy014 PMC6004785 29938232

[gbag165-B12] Glauber R. (2017). Gender differences in spousal care across the later life course. Research on Aging, 39, 934–959. 10.1177/0164027516644503 27193046

[gbag165-B13] Goldstein H. , Healy M. J. (1995). The graphical presentation of a collection of means. Journal of the Royal Statistical Society: Series A (Statistics in Society), 158, 175–177. 10.2307/2983411

[gbag165-B14] King McLaughlin J. , Greenfield J. C., Hasche L., De Fries C. (2019). Young adult caregiver strain and benefits. Social Work Research, 43, 269–278. 10.1093/swr/svz019

[gbag165-B15] Kreyenfeld M. , Bastin S. (2016). Reliability of union histories in social science surveys: Blurred memory, deliberate misreporting, or true tales? Advances in Life Course Research, 27, 30–42. 10.1016/j.alcr.2015.11.001

[gbag165-B16] Lacey R. E. , Xue B., McMunn A. (2022). The mental and physical health of young carers: A systematic review. The Lancet Public Health, 7, e787–e796. 10.1016/S2468-2667(22)00161-X(22)00161-X 36057277

[gbag165-B17] Larkina M. , Qian W., Yu W., Smith J. (2025). Cross-wave 2015–2019 Life History Mail Survey (LHMS): Harmonized and aggregated public data resource. https://hrsdata.isr.umich.edu/sites/default/files/documentation/data-descriptions/1739814830/LHMS1519_DD.pdf

[gbag165-B18] Lee C. , Glei D. A., Park S., Brown C. K., Weinstein M. (2026a). When family caregiving happens: Life-course timing and health outcomes in later life. The Journals of Gerontology, Series B: Psychological Sciences and Social Sciences, 81. 10.1093/geronb/gbag024 PMC13075644 41701143

[gbag165-B19] Lee C. , Zheng X., Park S. (2026b). The toll of caregiving: Life-course family care and disparities in physical functioning. The Journals of Gerontology, Series B: Psychological Sciences and Social Sciences, 81, gbaf246. 10.1093/geronb/gbaf246 PMC13591643 41352987

[gbag165-B20] Levine C. , Hunt G. G., Halper D., Hart A. Y., Lautz J., Gould D. A. (2005). Young adult caregivers: A first look at an unstudied population. American Journal of Public Health, 95, 2071–2075. 10.2105/AJPH.2005.067702 PMC1449485 16195506

[gbag165-B21] McCann J. J. , Hebert L. E., Bienias J. L., Morris M. C., Evans D. A. (2004). Predictors of beginning and ending caregiving during a 3-year period in a biracial community population of older adults. American Journal of Public Health, 94, 1800–1806. 10.2105/AJPH.94.10.1800 PMC1448537 15451753

[gbag165-B22] Macchioni Giaquinto A. , Jones A. M., Rice N., Zantomio F. (2022). Labor supply and informal care responses to health shocks within couples: Evidence from the UK. Health Economics, 31, 2700–2720. 10.1002/hec.4604 PMC9826460 36114626

[gbag165-B23] Miller K. E. M. , Hart J. L., Useche Rosania M., Coe N. B. (2024). Youth caregivers of adults in the United States: Prevalence and the association between caregiving and education. Demography, 61, 829–847. 10.1215/00703370-11383976 PMC11539003 38785364

[gbag165-B24] Miyawaki C. E. , Hooyman N. R. (2023). A systematic review of the literature on transnational caregiving: Immigrant adult children to ageing parents in home country. Journal of Family Studies, 29, 453–470. 10.1080/13229400.2021.1908908

[gbag165-B25] Moen P. , Robison J., Fields V. (1994). Women’s work and caregiving roles: A life course approach. Journal of Gerontology, 49, S176–S186. 10.1093/geronj/49.4.S176 8014401

[gbag165-B26] National Alliance for Caregiving, & AARP Public Policy Institute. (2015). *Caregiving in the United States: 2015 report*. Retrieved August 28, 2026, from https://www.aarp.org/content/dam/aarp/ppi/2015/caregiving-in-the-united-states-2015-report-revised.pdf

[gbag165-B27] National Alliance for Caregiving, & AARP Public Policy Institute. (2025). *Caregiving in the United States: 2025 report.* Retrieved August 28, 2026, from 10.26419/ppi.00373.001

[gbag165-B28] Pebley A. R. , Goldman N., Andrasfay T., Pratt B. (2021). Trajectories of physical functioning among older adults in the United States by race, ethnicity, and nativity: Examining the role of working conditions. PLoS One, 16, e0247804. 10.1371/journal.pone.0247804 PMC7968635 33730061

[gbag165-B29] Pinquart M. , Sörensen S. (2006). Gender differences in caregiver stressors, social resources, and health: An updated meta-analysis. The Journals of Gerontology, Series B: Psychological Sciences and Social Sciences, 61, P33–P45. 10.1093/geronb/61.1.P33 16399940

[gbag165-B30] Quiñones A. R. , Botoseneanu A., Markwardt S., Nagel C. L., Newsom J. T., Dorr D. A., Allore H. G. (2019). Racial/ethnic differences in multimorbidity development and chronic disease accumulation for middle-aged adults. PLoS One, 14, e0218462. 10.1371/journal.pone.0218462 PMC6576751 31206556

[gbag165-B31] Rote S. M. , Moon H. (2018). Racial/ethnic differences in caregiving frequency: Does immigrant status matter? The Journals of Gerontology, Series B: Psychological Sciences and Social Sciences, 73, 1088–1098. 10.1093/geronb/gbw106 27573991

[gbag165-B32] Settersten R. A. Jr. , Hägestad G. O. (1996). What’s the latest? Cultural age deadlines for family transitions. The Gerontologist, 36, 178–188. 10.1093/geront/36.2.178 8920087

[gbag165-B33] Taylor R. J. , Chatters L. M., Woodward A. T., Brown E. (2013). Racial and ethnic differences in extended family, friendship, fictive kin, and congregational informal support networks. Family Relations, 62, 609–624. 10.1111/fare.12030 PMC4116141 25089067

[gbag165-B34] Umberson D. , Olson J. S., Crosnoe R., Liu H., Pudrovska T., Donnelly R. (2017). Death of family members as an overlooked source of racial disadvantage in the United States. Proceedings of the National Academy of Sciences, 114, 915–920. 10.1073/pnas.1605599114 PMC5293066 28115712

