# Supplementary material for: Gender and racial/ethnic disparities in the life-course timing of first unpaid caregiving: a survival analysis approach
Source: J Gerontol B Psychol Sci Soc Sci. 2026 Aug 18;81(11):gbag165. doi: 10.1093/geronb/gbag165 (PMC13563413; doi:10.1093/geronb/gbag165)
Supplement: Supplementary file 1 [file gbag165_supplementary_data.docx]

***The Journals of Gerontology, Series B: Psychological Sciences and Social Sciences* Supplementary Material: Lee, Glei, Park, & Weinstein. Gender and racial/ethnic disparities in the life-course timing of first unpaid caregiving: A survival analysis approach.**

**TEXTS**

Text S1. Life History Mail Survey (LHMS) Sample, Selection and Sample Comparability

**Text S2.** Analytic Sample Case Selection Process

**Text S3. Setting the Lower Age Bound for Caregiving Onset at 10 and Handling Outliers**

Text S4. Sensitivity Analysis for 1^st^ Caregiving to Parent/Parent-in-Law

Text S5. Associations Between Age at First Caregiving Onset and Other Caregiving Characteristics Among Caregivers

Text S6. Retrospective Report of Caregiving and Issues related to Recall Bias.

**TABLES**

Table S1. Descriptive Statistics (Proportion or Means [SD]) for Analytic Sample by Subgroups (N=11,624)

Table S2. Characteristics 1^st^ Caregiving by Subgroups of Caregivers (N=3,073)

Table S3. Cumulative Risk by Age *t* of 1^st^ Caregiving to Parent/Parent-in law by Subgroups

Table S4. Cumulative Risk by Age *t* of 1^st^ Caregiving to Parent/Parent-in law by Subgroup, Censored at the Age When the Last Parent Was Known to be Alive

**FIGURES**

Figure S1. Analytic Sample Case Selection

Figure S2. Age-Specific Hazard of 1^st^ Unpaid Caregiving (Unadjusted)

Figure S3. Cumulative Risk of 1^st^ Caregiving to Parent/Parent-in law by Groups

Figure S4. Age-Specific Hazard of 1^st^ Caregiving to Parent/Parent-in law by Groups

Figure S5. Risk of 1^st^ Caregiving to Parent/Parent-in law over the Life Course by Gender, Censored at the Age When the Last Parent Was Known to be Alive

Figure S6. Risk of 1^st^ Caregiving to Parent/Parent-in law over the Life Course by Race/Ethnicity, Censored at the Age When the Last Parent Was Known to be Alive

**Text S1. Life History Mail Survey (LHMS) Sample, Selection and Sample Comparability**

The Life History Mail Survey (LHMS) was administered to selected HRS subsamples, not to the full HRS cohort. As described in the LHMS documentation (Larkina et al., 2025), selection varied across waves based on cohort membership, assignment to the Consumption and Activities Mail Survey (CAMS), language eligibility, previous LHMS participation, and prior response status. The 2015 LHMS was a short pilot survey administered only to eligible English-speaking respondents who were not assigned to CAMS. Beginning in 2017, the survey was expanded to include caregiving histories and Spanish-language questionnaires. Respondents who completed the 2015 LHMS were later given opportunities to complete the caregiving-history section through supplemental questionnaires in 2017 or 2019. Therefore, respondents who completed only the 2015 pilot survey but did not complete a later supplemental questionnaire were excluded from our analysis because caregiving histories were not available. The overall unweighted response rate for the cross-wave 2015–2019 LHMS was 60.8%. The LHMS can be linked to the longitudinal HRS core data using respondent identifiers.

To assess sample comparability, we used the HRS Tracker file (2022 version 1.0) to compare four groups: respondents who completed only the 2015 LHMS, respondents included in the 2017/2019 analytic sample, respondents who were selected for the LHMS but did not return the questionnaire, and respondents who were not selected for the LHMS. Because age and marital status vary by survey year, we focused on birth year, gender, race/ethnicity and nativity, and educational attainment.

The results suggest that the analytic sample is somewhat selective. Compared with respondents who were selected but did not return the questionnaire, respondents in the analytic sample were more likely to be female and to have higher education. The analytic sample also included a higher share of Non-Hispanic White respondents and a lower share of Non-Hispanic Black respondents compared with nonrespondents. Foreign-born Hispanic respondents were underrepresented in the 2015 pilot survey, likely because the 2015 survey was limited to English-speaking respondents, but they were better represented in the 2017/2019 analytic sample after Spanish-language questionnaires were introduced. Respondents who were not selected for the LHMS were from older birth cohorts, partly and possibly because the LHMS began in 2015 and was rolled out across HRS cohorts over subsequent waves, giving younger cohorts more opportunities to complete the survey. Overall, these patterns suggest that the findings from this study are restricted to this LHMS sample and may not generalize to the broader HRS population. This should be considered when interpreting the findings.

**Reference**

Larkina, M., Qian, W., Yu, W., & Smith, J. (2025). Cross-wave 2015–2019 Life History Mail Survey (LHMS): Harmonized and aggregated public data resource—Data description (Version 1.0). University of Michigan, Institute for Social Research. Health and Retirement Study.

**Text S2. Analytic Sample Case Selection Process**

We minimize sample selection by excluding only respondents who lack information on key variables or who fall outside the scope of our analysis.

Starting with the original sample of 2017 and 2019 LHMS respondents (n = 13,879), we first restricted the sample to those with valid caregiving information (i.e., whether they ever provided unpaid caregiving and the year the first episode began) and birth year, which are necessary to construct age at caregiving onset. This resulted in a sample of n = 12,607.

Second, because our analysis focuses on non-Hispanic Whites, non-Hispanic Blacks, and Hispanics, we excluded respondents from other racial/ethnic groups or those with missing race/ethnicity information (n = 548). In addition, we restricted the sample to respondents born during or before 1965 to ensure that individuals had reached at least midlife by the time of the LHMS interview, allowing us to capture caregiving experiences from early life through midlife or older ages. Respondents born in 1966 or later were therefore excluded (n=430). Finally, we removed five cases with missing information on the number of siblings across the HRS waves. Details of sample selection are now provided in Figure S1.

**Text S3: Setting the Lower Age Bound for Caregiving Onset at 10 and Handling Outliers**

Defining a minimum age for caregiving onset is not straightforward, as prior studies and national surveys have used varying thresholds. Existing research indicates that caregiving can begin at very young ages. A recent review of young carers (Lacey et al., 2021) documents caregiving onset as early as age 5, with ages 8–11 among the most commonly studied ranges and age 10 frequently used as a reference point. Consistent with this, U.S.-based national survey evidence (National Alliance for Caregiving & United Hospital Fund, 2005) shows that children aged 8–11 are capable of providing instrumental activities of daily living (IADLs), and in some cases basic activities of daily living (ADLs), particularly when co-residing with care recipients. In that report, over 90% of caregivers aged 8–11 provided at least one IADL, and a substantial share also reported involvement in ADLs.

At the same time, more conservative thresholds have been used in recent studies. For example, Miller et al. (2024) define youth caregiving beginning at age 15, and this threshold has been adopted in subsequent work using HRS LHMS (e.g., Chin et al., 2026). Taken together, these studies suggest that there is no single standard cutoff, and that the appropriate lower bound depends on the balance between capturing types of early caregiving experiences and ensuring measurement reliability.

In this study, we set the lower bound of caregiving onset at age 10, as the HRS LHMS captures caregiving activities that include IADLs (e.g., assistance with household tasks). This choice reflects both the empirical distribution of caregiving onset in our data and concerns about data quality at very young ages. Among our study sample (n = 11,624), 3,073 respondents reported at least one caregiving episode with sufficient information to calculate age at onset (derived from birth year and the reported start year of the episode). Of these, only eight cases reported caregiving onset before age 10. Specifically, seven respondents reported onset ages of 0, 3, 4, 6, 7, 8, or 9 years, and one respondent reported a caregiving start year that preceded their birth year. Given the extremely small number of such cases and the likelihood of recall or reporting error, we treated these observations as outliers.

For these eight outliers, we reassigned caregiving onset to the start age of their second reported caregiving episode, which resulted in onset ages ranging from 15 to 47. This approach allows us to retain these respondents in the analytic sample while avoiding reliance on implausible or low-reliability early-life reports. The eight outliers included four non-Hispanic Whites and four non-Hispanic Blacks.

To assess the robustness of our findings to this decision, we conducted sensitivity analyses using alternative specifications excluding these cases entirely. Given the extremely small number of outliers, our findings remained unchanged even when these cases were excluded.

**Reference**

Chin, J. A., & Lin, I. F. (2026). Unpaid caregiving across the lifespan and health outcomes in later life.

Advances in Life Course Research, 67, 100722.

Lacey, R. E., Xue, B., & McMunn, A. (2022). The mental and physical health of young carers: A systematic review. *The Lancet Public Health*, *7*(9), e787-e796.

Miller, K. E. M., Hart, J. L., Useche Rosania, M., & Coe, N. B. (2024). Youth caregivers of adults in the United States: Prevalence and the association between caregiving and education. *Demography*, *61*(3), 829–847

National Alliance for Caregiving & United Hospital Fund. (2005). *Young Caregivers in the U.S.: Findings from a National Survey*. Bethesda, MD: National Alliance for Caregiving.

**Text S4. Sensitivity Analysis for 1^st^ Caregiving to Parent/Parent-in-Law**

Respondents identified their relationship to the first individual to whom they provided care, including parent or parent-in-law, spouse or partner, biological/adopted/stepchild, and others. Parents or parents-in-law were the most common first care recipients (58%; Table S2); thus, sensitivity analyses focused on parental/parental-in-law caregiving onset.

In our main analysis, individuals who did not provide unpaid caregiving (N=8,551) were censored at the age when they completed the Life History Mail Survey (LHMS). However, if a respondent’s parent(s) died before that time, this censoring strategy could bias the results. To address this concern, we used information from the HRS Core survey (parents’ ages and years of death) to censor respondents at the last year in which at least one parent was known to be alive.

Among 11,624 respondents, 322 (2.7%) had both parents deceased but were missing either the dates of death (n = 279) or the vital status of both parents (n = 43). Missingness was somewhat higher among Non-Hispanic Black (3.1%), U.S.-born Hispanic (2.9%), and foreign-born Hispanic (4.0%) respondents compared with Non-Hispanic Whites (2.5%). We also removed respondents who lost their both parents before age 10 (n=25). Thus, our analytic sample for this sensitivity analysis was 11,277 respondents.

Because censoring was based on the last year a parent was known to be alive rather than the LHMS interview age, approximately 65% of respondents were censored earlier. Using this revised censoring strategy, we re-estimated the cumulative hazard and age-specific hazard of first caregiving for a parent or parent-in-law. It is important to note that the HRS does not provide information on the vital status or death dates of parents-in-law; therefore, we could not distinguish caregiving for parents from caregiving for parents-in-law in this sensitivity analysis, and some bias may remain.

Nonetheless, the overall conclusions (See Table S3 and S4, Figure S3-S6) and were consistent with those from our primary analysis based on censoring at the LHMS interview age.

**Text S5. Associations Between Age at First Caregiving Onset and Other Caregiving Characteristics Among Caregivers**

There is an inverse association between age at caregiving onset and the duration of the first caregiving episode (*r* = −.40). That is, individuals who enter caregiving earlier in life tend to experience longer durations and are more likely to have additional caregiving episodes over the life course (see below).

| **Age at Caregiving Onset** | **Aged 10-24** | **Aged 25-34** | **Aged 35-44** | **Aged 45-54** | **Aged 55-64** | **Aged 65+** |
| --- | --- | --- | --- | --- | --- | --- |
| Duration of 1^st^ Caregiving, Mean (SD) | 17.4 (15.39) | 13.33 (12.83) | 8.50 (8.36) | 6.03 (5.51) | 4.64 (4.40) | 3.90 (3.31) |
| Repeated caregiving, % | 43.3 | 41.2 | 37.2 | 27.6 | 17.4 | 8.21 |

Note. Age categories for the first onset of caregiving are based on prior work (Lee et al., 2026).

**Reference**

Lee, C., Glei, D. A., Park, S., Brown, C. K., & Weinstein, M. (2026). When Family Caregiving Happens: Life-Course Timing and Health Outcomes in Later Life. *The Journals of Gerontology, Series B: Psychological Sciences and Social Sciences*, gbag024.

**Text S6. Retrospective Report of Caregiving and Issues related to Recall Bias.**

The unpaid caregiving measure is derived from the HRS LHMS, which uses a life history calendar approach to collect retrospective information on a wide range of salient life events, including schooling, employment, partnerships, and caregiving episodes. **The LHMS design** incorporates several features known to enhance recall, such as chronological reconstruction, the use of age- and role-based cues, and anchoring to salient life events, which together facilitate more accurate reporting (Belli, 1998; Freedman et al., 1988; Axinn et al., 1999). In addition, respondents are explicitly encouraged to consult family members or personal records when completing the survey (e.g., “*This survey is not meant to be a test of your memory… You may find it useful to consult your spouse, another family member, or personal files, photos, or notes”*), which may further reduce recall error.

Prior research suggests that life history calendar methods improve the quality of retrospective reports and that salient life events are recalled with reasonable accuracy (Axinn et al., 1999; Kreyenfeld & Bastin, 2016). Because the LHMS focuses on caregiving episodes lasting six months or longer, it is more likely to capture sustained and meaningful caregiving experiences rather than short-term or routine assistance. **Transitions into unpaid caregiving of this duration are therefore more likely to be recalled accurately**, as they often represent salient life events that may spill over into other domains of individuals’ lives, such as schooling, employment, or family formation.

Nonetheless, some underreporting—particularly with respect to the timing of onset—is possible. The direction of bias, however, is not straightforward. On one hand, caregiving episodes that occurred earlier in life may be more susceptible to forgetting, especially if they involved less intensive or more instrumental forms of support **or if respondents experience memory decline**. On the other hand, first caregiving experiences—such as caring for a family member with a serious illness or disability—may represent major life transitions and thus be more salient and memorable, making them less likely to be missed. Taken together, any recall bias is likely to vary by the timing and salience of caregiving experiences, rather than systematically biasing estimates in a single direction

**References**

Axinn, W. G., Pearce, L. D., & Ghimire, D. (1999). Innovations in life history calendar applications. Social Science Research, 28(3), 243–264.

Belli, R. F. (1998). The structure of autobiographical memory and the event history calendar: Potential improvements in the quality of retrospective reports in surveys. Memory, 6(4), 383–406.

Belli, R. F., Shay, W. L., & Stafford, F. P. (2001). Event history calendars and question list surveys: A direct comparison of interviewing methods. *Public Opinion Quarterly*, *65*(1), 45-74.

Conway, M. A., & Pleydell-Pearce, C. W. (2000). The construction of autobiographical memories in the self-memory system. Psychological Review, 107(2), 261–288.

Freedman, D., Thornton, A., Camburn, D., Alwin, D., & Young-DeMarco, L. (1988). The life history calendar: A technique for collecting retrospective data. Sociological Methodology, 18, 37–68.

Kreyenfeld, M., & Bastin, S. (2016). Reliability of union histories in social science surveys: blurred

memory, deliberate misreporting, or true tales? *Advances in Life Course Research*, *27*, 30-42.

**Table S1. Descriptive Statistics (Percentage or Means [SD]) for Analytic Sample by Subgroups (N=11,624)**

| **Variables** | **Men**  **(N=4816)** | **Women**  **(N=6808)** |  | **Non-Hispanic White**  **(N=8009)** | **Non-Hispanic**  **Black**  **(N=2153)** | **U.S.-born Hispanic**  **(N=588)** | **Foreign-born Hispanic**  **(N=874)** |
| --- | --- | --- | --- | --- | --- | --- | --- |
| Any Unpaid Caregiving, % | 18.0 | 32.4 |  | 27.9 | 25.6 | 23.6 | 17.0 |
| Parental(in-law) Care, % | 11.3 | 21.0 |  | 18.3 | 15.5 | 16.3 | 8.9 |
|  |  |  |  |  |  |  |  |
| Women, % |  |  |  | 57.7 | 63.6 | 56.6 | 55.7 |
|  |  |  |  |  |  |  |  |
| *Race/Ethnicity* |  |  |  |  |  |  |  |
| Non-Hispanic White, % | 70.4 | 67.8 |  | - | - | - | - |
| Non-Hispanic Black, % | 16.3 | 20.1 |  | - | - | - | - |
| U.S.-born Hispanic, % | 5.0 | 4.9 |  | - | - | - | - |
| Foreign-born Hispanic, % | 8.0 | 7.2 |  | - | - | - | - |
|  |  |  |  |  |  |  |  |
| Age, Mean (SD) | 69.8 (10.0) | 69.7 (10.4) |  | 71.3 (10.4) | 66.6 (9.0) | 66.6 (9.0) | 65.5 (9.0) |
|  |  |  |  |  |  |  |  |
| Number of Siblings, Mean (SD) | 3.4 (2.6) | 3.6 (2.7) |  | 2.8 (2.1) | 4.8 (3.1) | 5.0 (3.2) | 5.6 (3.2) |
|  |  |  |  |  |  |  |  |
| *Cohorts* |  |  |  |  |  |  |  |
| ≤ 1941 (HRS or older), % | 29.9 | 29.0 |  | 34.9 | 17.4 | 19.1 | 15.3 |
| 1942-53 (War babies/Early boomers), % | 33.2 | 33.3 |  | 33.8 | 33.9 | 30.6 | 28.8 |
| 1954-65 (Mid/Late boomers), % | 36.9 | 37.7 |  | 31.4 | 48.7 | 50.3 | 55.8 |

*Notes.* Number of siblings was derived from the maximum available information across HRS waves, 1992–2020.

**Table S2. Characteristics of 1^st^ Caregiving by Subgroups of Caregivers**

|  | **Total**  **(N=3073)** | **Men**  **(N=869)** | **Women**  **(N=2204)** |  | **Non-Hispanic White**  **(N=2,233)** | **Non-Hispanic**  **Black**  **(N=552)** | **U.S.-born Hispanic**  **(N=139)** | **Foreign-born Hispanic**  **(N=149)** |
| --- | --- | --- | --- | --- | --- | --- | --- | --- |
| Relationship to 1^st^ Care-recipient (N=3,073) |  |  |  |  |  |  |  |  |
| Parent/Parent-in-law, % | 57.6 | 58.7 | 57.1 |  | 58.7 | 54.0^b^ | 62.6 | 49.0^b^ |
| Spouse or partner,% | 17.1 | 18.6 | 16.5 |  | 18.3 | 12.0 | 14.4 | 20.1^b^ |
| Biological, adopted, or step-child,% | 8.3 | 8.1 | 8.4 |  | 7.8 | 9.6 | 5.0 | 14.8^b^ |
| Other,% | 14.5 | 12.2 | 15.4 |  | 12.8 | 21.6 | 15.1^b^ | 12.8 |
| Multiple recipients,% | 1.4 | 0.9 | 1.6 |  | 1.5 | 0.5^b^ | 2.9 | 1.3 |
| Unknown, % | 1.1 | 1.5 | 1.0 |  | 0.9 | 2.4 | 0 | 2.0 |
|  |  |  |  |  |  |  |  |  |
| Onset age at 1^st^ Caregiving (N=3073), Mean (SD) | 49.22 (14.21) | 49.59 (14.68) | 49.06  (14.03) |  | 50.10 (14.19) | 47.46  (13.66) | 45.83  (13.75) | 45.74  (15.58) |
|  |  |  |  |  |  |  |  |  |
| Duration of 1^st^ Caregiving (N=2,773)* , Mean (SD) | 7.22 (8.29) | 7.84 (7.95)^a^ | 6.98(7.95)^a^ |  | 7.17 (8.20) | 7.22  (8.37) | 6.98  (8.21) | 8.23  (8.10) |
|  |  |  |  |  |  |  |  |  |
| Multiple caregiving episodes over the life course (N=3,073), % | 26.7 | 19.2 ^a^ | 29.58 ^a^ |  | 26.7 | 27.0 | 28.8 | 22.2 |
|  |  |  |  |  |  |  |  |  |

*Note.* Unknown = Respondents reported that they provided unpaid caregiving and the period of care, but did not report the care-recipient. *The total N for the duration of the first caregiving episode is reduced to 2,773 due to missing information on the end year of 1^st^ caregiving. ^a^= Gender differences remain statistically significant at p < .05 after controlling for baseline age. ^b^= comparison racial/ethnic groups differ significantly from non-Hispanic Whites at p <.05 after adjusting for baseline age.

**Table S3. Cumulative Risk by Age *t* of 1^st^ Caregiving to Parent/Parent-in-Law by Subgroups**

|  | **Cumulative Risk (CI)** | | | | | | |  | **Ratio of Cumulative Risk relative to Reference Group** | | | | |
| --- | --- | --- | --- | --- | --- | --- | --- | --- | --- | --- | --- | --- | --- |
| **Age at *t*** | **Men** | **Women** |  | **NHW** | **NHB** | **USH** | **FBH** |  | **Women^a^** | **NHB^b^** | **USH^b^** | **FH^b^** | **USH^c^** |
|  |  |  |  |  |  |  |  |  |  |  |  |  |  |
| ***20^#^*** | .004 | .005 |  | .005 | .005 | .005 | .005 |  | 1.25 | 1.04 | 1.13 | 1.02 | 1.00 |
|  | (.003-.005) | (.004-.006) |  | (.004-.006) | (.003-.007) | (.002-.011) | (.002-.009) |  |  |  |  |  |  |
|  |  |  |  |  |  |  |  |  |  |  |  |  |  |
| ***30*** | .011 | .017 |  | .014 | .017 | .024 | .010 |  | 1.56** | 1.25 | 1.75* | 0.75 | 2.40* |
|  | (.009-.013) | (.015-.020) |  | (.012-.016) | (.014-.021) | (.017-.034) | (.007-.016) |  |  |  |  |  |  |
|  |  |  |  |  |  |  |  |  |  |  |  |  |  |
| ***40*** | .027 | .049 |  | .038 | .041 | .061 | .033 |  | 1.83** | 1.07 | 1.61* | 0.85 | 1.85* |
|  | (.023-.030) | (.045-.052) |  | (.004-.041) | (.036-.047) | (.049-.076) | (.026-.041) |  |  |  |  |  |  |
|  |  |  |  |  |  |  |  |  |  |  |  |  |  |
| ***50*** | .058 | .119 |  | .096 | .094 | .105 | .059 |  | 2.06** | 0.98 | 1.10 | 0.61* | 1.78* |
|  | (.053-.063) | (.113-.125) |  | (.092-.101) | (.086-.103) | (.089-.125) | (.049-.071) |  |  |  |  |  |  |
|  |  |  |  |  |  |  |  |  |  |  |  |  |  |
| ***60*** | .102 | .207 |  | .172 | .152 | .166 | .089 |  | 2.03** | 0.89* | 0.96* | 0.51* | 1.87* |
|  | (.096-.109) | (.199-.215) |  | (.166-.179) | (.142-.164) | (.144-.190) | (.076-.103) |  |  |  |  |  |  |
|  |  |  |  |  |  |  |  |  |  |  |  |  |  |
| ***N at risk***  ***at age 20*** | 4800 | 6781 |  | 7980 | 2145 | 586 | 871 |  |  |  |  |  |  |

*Notes.* ^#^Although the estimation begins at age 10, we reported results starting at age 20 due to sparse events at younger ages. NHW= non-Hispanic Whites, NHB=non-Hispanic Blacks, USH=U.S.-born Hispanics, FH=Foreign-born Hispanics. CI= confidence interval. Cumulative hazard at age t is estimated using the Nelson–Aalen estimator. Bolded text indicates significant differences from the reference group. Reference groups: ^a^men for gender, ^b^non-Hispanic Whites for race/ethnicity groups, ^c^Foreign-born Hispanics for U.S. born Hispanic. Significance is determined by non-overlapping confidence intervals (in parentheses) under the Goldstein–Healy rule.

**p <* .05*,* ***p < .*10.

**Table S4. Cumulative Risk by Age *t* of 1^st^ Caregiving to Parent/Parent-in-Law by Subgroups, Censored at the Age When the Last Parent Was Known to be Alive**

|  | **Cumulative Risk (CI)** | | | | | | |  | **Ratio of Cumulative Risk relative to Reference Group** | | | | |
| --- | --- | --- | --- | --- | --- | --- | --- | --- | --- | --- | --- | --- | --- |
| **Age at *t*** | **Men** | **Women** |  | **NHW** | **NHB** | **USH** | **FBH** |  | **Women^a^** | **NHB^b^** | **USH^b^** | **FH^b^** | **USH^c^** |
|  |  |  |  |  |  |  |  |  |  |  |  |  |  |
| ***20^#^*** | .004 | .005 |  | .004 | .005 | .005 | .005 |  | 1.20 | 1.29 | 1.26 | 1.14 | 1.00 |
|  | (.003-.006) | (.004-.006) |  | (.003-.005) | (.004-.008) | (.003-.011) | (.003-.009) |  |  |  |  |  |  |
|  |  |  |  |  |  |  |  |  |  |  |  |  |  |
| ***30*** | .012 | .019 |  | .015 | .020 | .025 | .011 |  | 1.54** | 1.31 | 1.70* | 0.73 | 2.50* |
|  | (.010-.015) | (.016-.021) |  | (.013-.017) | (.016-.024) | (.018-.036) | (.007-.017) |  |  |  |  |  |  |
|  |  |  |  |  |  |  |  |  |  |  |  |  |  |
| ***40*** | .030 | .055 |  | .043 | .048 | .068 | .032 |  | 1.80** | 1.10 | 1.57* | 0.73* | 2.13* |
|  | (.027-.034) | (.051-.059) |  | (.040-.046) | (.042-.054) | (.055-.084) | (.024-.041) |  |  |  |  |  |  |
|  |  |  |  |  |  |  |  |  |  |  |  |  |  |
| ***50*** | .073 | .143 |  | .116 | .120 | .126 | .066 |  | 1.97** | 1.04 | 1.09 | 0.57* | 1.90* |
|  | (.067-.079) | (.136-.151) |  | (.110-.121) | (.110-.132) | (.107-.149) | (.055-.080) |  |  |  |  |  |  |
|  |  |  |  |  |  |  |  |  |  |  |  |  |  |
| ***60*** | .149 | .300 |  | .246 | .233 | .233 | .122 |  | 2.00** | 0.95* | 0.95* | 0.50* | 1.90* |
|  | (.139-.159) | (.286-.311) |  | (.236-.255) | (.216-.252) | (.201-.270) | (.103-.144) |  |  |  |  |  |  |
|  |  |  |  |  |  |  |  |  |  |  |  |  |  |
| ***N at risk***  ***at age 20*** | 4646 | 6514 |  | 7735 | 2033 | 562 | 830 |  |  |  |  |  |  |

*Notes.* ^#^Although the estimation begins at age 10, we originally reported results starting at age 20 due to sparse events at younger ages. NHW= non-Hispanic Whites, NHB=non-Hispanic Blacks, USH=U.S.-born Hispanics, FH=Foreign-born Hispanics. CI= confidence interval. Cumulative hazard at age t is estimated using the Nelson–Aalen estimator. Bolded text indicates significant differences from the reference group. Reference groups: ^a^men for gender, ^b^non-Hispanic Whites for race/ethnicity groups, ^c^Foreign-born Hispanics for U.S. born Hispanic. Significance is determined by non-overlapping confidence intervals (in parentheses) under the Goldstein–Healy rule.

**p <* .05*;* ***p < .*10.

**Figure S1. Analytic Sample Case Selection**

**Figure S2. Age-Specific Hazard of 1^st^ Unpaid Caregiving (Unadjusted)**

**Figure S3. Risk of 1^st^ Caregiving to Parent/Parent-in law over the Life Course by Gender**

**(A) Cumulative Hazard**

**(B) Age-Specific Hazard**

*Notes.* Cumulative hazard is estimated using the Nelson–Aalen estimator. Age-specific hazard estimates are derived from Cox models controlling for gender, race/ethnicity, birth cohort, age at LHMS interview, and the maximum number of siblings observed across HRS waves (1992–2020)

**Figure S4. Risk of 1^st^ Caregiving to Parent/Parent-in law over the Life Course by Race/Ethnicity**

**(A) Cumulative Hazard**

**(B) Age-Specific Hazard**

Notes. Cumulative hazard is estimated using the Nelson–Aalen estimator. Age-specific Hazard estimates are derived from Cox models controlling for gender, race/ethnicity, birth cohort, age at the Life History Mail Survey, and the maximum number of siblings observed across HRS waves (1992–2020). To allow for non-proportional hazards, the model includes interactions between age and race/ethnicity.

**Figure S5. Risk of 1^st^ Caregiving to Parent/Parent-in law over the Life Course by Gender, Censored at the Age When the Last Parent Was Known to be Alive**

**(A) Cumulative Hazard**

**(B) Age-Specific Hazard**

*Notes.* Cumulative hazard is estimated using the Nelson–Aalen estimator. Age-specific hazard estimates are derived from Cox models controlling for gender, race/ethnicity, birth cohort, age at LHMS interview, and the maximum number of siblings observed across HRS waves (1992–2020).

**Figure S6. Risk of 1^st^ Caregiving to Parent/Parent-in law over the Life Course by Race/Ethnicity, Censored at the Age When the Last Parent Was Known to be Alive**

**(A) Cumulative Hazard**

**(B) Age-Specific Hazard**

Notes. Cumulative hazard is estimated using the Nelson–Aalen estimator. Age-specific Hazard estimates are derived from Cox models controlling for gender, race/ethnicity, birth cohort, age at the Life History Mail Survey, and the maximum number of siblings observed across HRS waves (1992–2020). To allow for non-proportional hazards, the model includes interactions between age and race/ethnicity.
